# Interaction-Induced Structural Transformations in Polysaccharide and Protein-Polysaccharide Gels as Functional Basis for Novel Soft-Matter: A Case of Carrageenans

**DOI:** 10.3390/gels8050287

**Published:** 2022-05-06

**Authors:** Olga N. Makshakova, Yuriy F. Zuev

**Affiliations:** 1 Kazan Institute of Biochemistry and Biophysics, FRC Kazan Scientific Center of RAS, Lobachevsky Str., 2/31, 420111 Kazan, Russia; zuev@kibb.knc.ru; 2 A. Butlerov Chemical Institute, Kazan Federal University, Kremlevskaya 18, 420008 Kazan, Russia

**Keywords:** protein–polysaccharide gels, κ-carrageenan, galactan, structure, crosslinking, FTIR-spectroscopy, molecular modelling, novel matter

## Abstract

Biocompatible, nontoxic, and biodegradable polysaccharides are considered as a promising base for bio-inspired materials, applicable as scaffolds in regenerative medicine, coatings in drug delivery systems, etc. The tunable macroscopic properties of gels should meet case-dependent requirements. The admixture of proteins to polysaccharides and their coupling in more sophisticated structures opens an avenue for gel property tuning via physical cross-linking of components and the modification of gel network structure. In this review recent success in the conformational studies of binary protein–polysaccharide gels is summarized with the main focus upon carrageenans. Future perspectives and challenges in rational design of novel polysaccharide-based materials are outlined.

## 1. Introduction

Polysaccharides are biocompatible nontoxic natural polymer compounds. They are widely used as functional soft-matter for exploration in the food industry, drug delivery, regenerative medicine, and other biomedical research and applications [1,2,3,4,5,6,7,8]. Polysaccharides are widely spread in bacterium, plant, and animal nature: produced by algae (alginate and carrageenan), plants (cellulose, pectin, and guar gum), bacterium (dextran and chitin) and animals (hyaluronan, chondroitin, and heparin). Polysaccharides with diversity of chemical modification represent a wide range of molecular weights. This work is devoted to carrageenans, one of the representatives of galactans. Galactans are a unique class of natural polysaccharides, distributed in plants (red algae, lupine seeds, citrus peel, etc.), also found in animals and microorganisms. We chose this polysaccharide family to review the interaction-induced structural transformations in polysaccharides and protein–polysaccharide gels as functional soft-matter because of our long-running deep interest and diverse experience in galactans [9,10,11,12].

**Figure 1:**
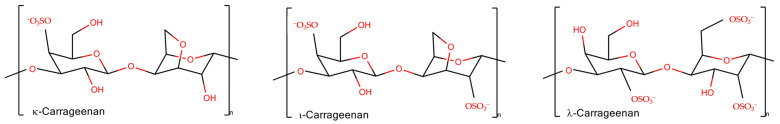
The schematic representation of disaccharide units of carrageenans.

Carrageenans are linear, sulfated, hydrophilic non-toxic polysaccharides from red algae. Their molecules consist of galactose repeat units, one ring per disaccharide can be (3,6)-anhydro, connected by alternating α-(1,3)- and β-(1,4)-glycosidic links with a variable proportion of sulfate groups at different positions [13]. There are different types of carrageenans (are widely used in technologic developments), carrying from one to three sulfate groups per monomeric unit of polymer (Figure 1). Among natural galactans the carrageenans are ideal candidates for different applications in wound healing and drug delivery [14]. Together with other sulfated polysaccharides [15], they are often considered as mimetics of glycosaminoglycans (GAG), the matrix polysaccharides of mammals, and have a potential to be used for tissue engineering [16]. In addition, the application of carrageenans is omnipresent in dairy products due to their strong interaction with food proteins [17].

Gels, one of the essential states of polysaccharides, represent a three-dimensional crosslinked polymer network immersed in a confined continuous fluid phase. If the fluid totals more than 90% of volume, it is referred to as hydrogel in the case of water or organogel in the case of organic fluid. In this review we examine only aqueous systems, thus we can define hydrogels as three-dimensional, hydrophilic polysaccharide or protein–polysaccharide networks that are able to retain a large amount of water within their structure without dissolving [18]. Gels are classified as chemical or physical depending on the cross-linker’s nature [19]. In the first case, the polymeric chains are held together by covalent chemical bonds. Mostly technologically advanced are physical hydrogels, which are typical of many natural biopolymers, in which a three-dimensional polymer network exists owing to the mechanical weave of the polymer molecules and/or stabilized by intermolecular interactions, including ionic bridges, hydrogen bonding, and hydrophobic interactions.

During the last decades the new trends in gel formation comprise stimuli-responsive gels, cell-compatible gels, and self-healing gels [20]. The usage of co-gelators is one of the engineering procedures to improve the gel strength, melting, and gelling temperatures and alter the physicochemical properties of the engineered biomedical or biotechnological formulations in the admixture of co-gelators. The admixture of proteins to polysaccharides opens a broad avenue for tuning gel properties via the regulation of a sophisticated distribution of components and/or their physical cross-linking that modifies both the gel network structure and properties [21]. The modulation of protein–polysaccharide gel properties is used to control texture, taste, and stability of food products [22]. For example, protein–polysaccharide co-gelling is used in meat products, i.e., to improve the texture and water holding capacity of hams [23] or sausages [24], and in milk products [25,26] to tune the viscosity of whipped cream [27] or to improve the ease of grating and slicing of cheese [28,29].

In accordance with the compatibility conditions, the interactions of biopolymers in aqueous mixtures can result in the formation of polyelectrolyte complexes [30], which have specific effects on the gelling in mixed systems. The phenomena of polymer coacervation and segregation became a topic of research at the beginning of the last century [31]. Aspects of coacervation between proteins and anionic polysaccharides are summarized in the reviews [32,33]. However, despite a rising number of studies on gel properties, the detailed information about conformational aspects of protein–polysaccharide adjustment upon their complexation is rather scarce. In particular, this is due to the methodological difficulties to probe conformation of interacting biopolymers in the soft matter condition. In this review the recent achievements in conformational studies of carrageenans in polysaccharide–protein gels are summarized. The review is organized as follows: in the first part, the types of chain crosslinking in polysaccharide gels are briefly discussed, in the second part, the different types of interactions in protein–polysaccharide gels driven by segregative and associative processes are summed up and then some case examples are given for the illustration of mutual influence on protein and polysaccharide conformations upon associative electrostatic interactions. As a conclusion, the future perspectives and challenges in rational design of novel polysaccharide-based materials are outlined.

## 2. Crosslinking in Polysaccharide Gels

The molecular weight of polysaccharides ranging from several hundred thousand to millions of Daltons allows them to form gels at concentrations lower than those required for protein gelation. Usually, less than 1 mass% of polysaccharide is necessary for its gelation via various intermolecular interactions [17]. Gel formation may occur via the long chain entanglement and formation of ordered structures in the junction zones. The packing of chains in junction zones is generally accompanied with the structuring of flexible polysaccharide chains and results in the increase of gel elasticity, whilst the simple entanglement contributes to the gel viscosity. Several types of chain–chain interactions in polysaccharide gels upon chain ordering have been proposed.

### 2.1. Chain Ordering in Carrageenans

Thermoreversible gels are formed by ι- and κ-carrageenans. The gel melts above a certain temperature and becomes firm when cooled. The firmness of the gel versus temperature may reveal hysteresis being dependent on the process direction. The melting temperature depends on the salt present, that is the type and concentration of counterions. The presence of two sulfate groups per one disaccharide in ι-carrageenan requires divalent cations to screen the repulsion of approaching chains and make a bridge between them stabilizing the gel formation. The κ-carrageenan needs monovalent cations, like K^+^, and to a lesser extent divalent cations. For λ-carrageenan, its gelation was reported only in the case of the presence of Fe^3+^ [34]. More detailed information about carrageenan gel rheology can be found in recent reviews on this topic [35,36].

The rheological properties are coupled with structural transitions of the carrageenan chains. The secondary structure changes from the coil to the helix state upon gelling. It still remains a matter of debate if carrageenans form single [37,38] or intertwined double helices [39,40].

The double helix formation with further cation mediated association in stacks is a widely accepted model for the junction zones in carrageenan gels. The double helix formation is supported by a large amount of data [17] including X-ray diffraction [39,41] and ultrasonic relaxation [42]. X-ray diffraction on crystalline ι-carrageenan revealed a parallel three-fold double helix with pitch around 26 Å [43]. Although experimental data for κ-carrageenan cannot be interpreted in terms of geometry [34], the modelling suggests a three-fold double helix with pitch of 25 Å, where both parallel and antiparallel chain orientations are possible [44]. A similar 25 Å pitch of helix was reported for λ-carrageenan [34]. Wide-angle X-ray diffraction on carrageenan solids packed in a form of membranes showed double-helical structure for ι- and κ-carrageenans and a single-helical one for λ-carrageenan [45]. Computer modelling favored a single helix [46].

Using high-resolution AFM microscopy, Schefer et al. [38,47] provided direct evidence of single helix formation in ι- and κ-carrageenans, similar to some other polysaccharides [48]. The monovalent ion-induced coil-helix transition is coupled with non-monotonic tendency of chain stiffening with persistent length variations from 10 to 20 nm [38]. In contrast, in the presence of NaCl, λ-carrageenan retains a random coil conformation [38]. Single helices may laterally associate into supercoils in the fashion of tertiary structure of proteins or/and to coil-coiled helices similar to quaternary structures and after this they can form supramolecular gels [49].

**Figure 2:**
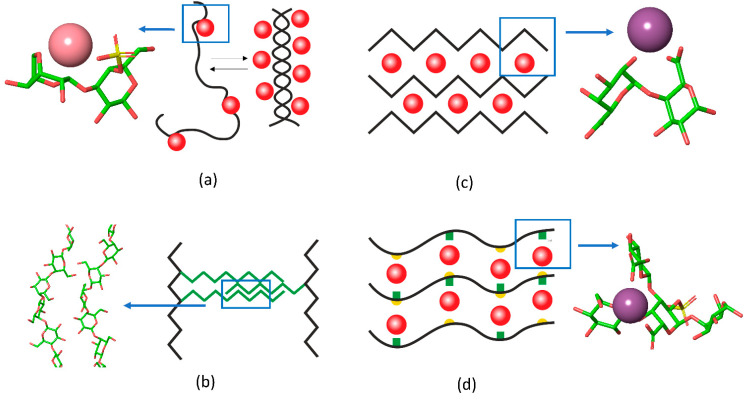
The schematic representation of interchain junctions and the atomistic representation of (**a**) key interactions in carrageenan, (**b**) 1,4-β-galactan in RG-I, (**c**) pectin gels, and (**d**) in Infernan gel. In the schemes, cations are shown as red balls, side chains are given in green, the location of sulfate groups in Infernan is indicated by yellow semicircles. K^+^ in the complex with κ-carrageenan is shown in pink and Ca^2+^ in complex with pectin and Infernan is shown in violet.

Recently, our group applied Fourier transform infrared (FTIR) spectroscopy to provide deeper insights into the coupling of ion binding with coil-helix transitions in κ-carrageenan upon the temperature-induced formation and melting of gels [10]. Because of pronounced overlapping of spectral bands, which is typical for polysaccharides, the set of spectra, recorded along the perturbation coordinate (i.e., temperature), was decomposed into orthogonal variants. Based on the splitting of the ν_as_SO_3_ complex band (1300–1200 cm^−1^) the orthogonal variants were attributed to sulfate groups in the free state and with weakly or strongly bound ions. Two orthogonal components in the region of skeletal vibrations (1200–1000 cm^−1^), interchanging in a cooperative fashion along the temperature, were the hallmark of helical and coil conformation. Further analysis coupled with quantum chemical calculations revealed the following sequence of events [10]: (1) upon cooling down the strong K^+^ binding becomes prevalent over weak contacts, (2) the strong K^+^ binding requires the neocarrabiose units to be in a conformation determined by the values of the phi and psi angles as those in the helix; at this stage the “seeds” of helical conformation are stabilized by bound potassium ions, (3) the further cooling of the system results in the full chain conversion to the helix conformation (Figure 2a).

The FTIR-spectral patterns of helix and coil conformations of κ-carrageenan can be used for the in situ detection of the polysaccharide conformational state in protein–carrageenan gels, which is illustrated in Section 3.

### 2.2. 1,4-β-Galactan Crosslinking

Another type of gel formation via galactan chains was reported recently for rhamnogalacturonan I (RG-I), which is usually present as a hairy region of the pectin molecule. The RG-I from gelatinous fibers of flax, which does not have homogalacturonan fragments and is branched with long 1,4-β-galactan side chains, can form physical gels under microwave irradiation [12]. In this type of gel, the RG-I molecules are entangled by galactan side chains. Interestingly, the 2D-correlation FTIR spectroscopy [9] together with molecular modelling [11] showed that in such gels 1,4-galactan has a regular helical structure, the side chains interact in a side-by-side antiparallel fashion (Figure 2b).

### 2.3. Egg-Box Assembly in Pectins and Alginates

A famous example of polysaccharide chain fine-tuning via ion cross-linking in gel junction zones is the packing of alginates and pectins in the form of an egg-box. The gelation of alginate, composed of (1→4)- linked α-L-guluronic and β-D-mannuronic residues of varying sequence, takes place under mild conditions with the sol-gel transition independent of temperature. Divalent cations induce chain–chain association by means of physical cross-linkage of guluronic acid blocks of neighboring alginate molecules, known as the “egg-box model” [50]. Another example is the homogalacturonan, a major domain of the pectin molecule, built up by the (1→4)- linked α-D-galacturonate residues. The GalpA residues offer their carboxylate groups for the ion coordination in the form of an egg box [51,52]. A recent review sums up the differences between pectin and alginate structure of the egg-box [53]. The progression of such a structure results in the formation of firm bunches of polysaccharide chains (Figure 2c).

A novel type of side-by-side chain interactions mediated by calcium ions has been recently proposed for Infernan, a capsular polysaccharide from the deep-sea bacterium *Alteromonas infernus*. Infernan is a branched polysaccharide, whose backbone consists of repeating units [→4)-β-D-Glc*p*-(1→4)-α-D-Gal*p*A-2S-(1→4)-α-D-Gal*p*-(1→]. Each galacturonate residue in the backbone bears a sulfate group at the 2d position and a side chain at the 3d position. In the side chain, which is also branched, two glucuronate residues are attached to α-D-GalpA of the backbone. Molecular modelling unraveled the energetically favorable conformation in which the negatively charged groups in the backbone and in the sidechain come to such proximity as to become capable to accommodate the calcium in a cage. The adjacent chain, interacting with the cage-forming one, completes the calcium coordination up to six [54] (Figure 2d).

The description of some other types of gel-forming polysaccharides and their structure can be found in the excellent reviews [6,55] including those focusing on pectins [56], nanocellulose, [57] chitosan gelation [58], and algal polysaccharides [5].

The chain stiffness of a polymer influences its properties and its capability to interact with other molecules [59]. Thus, the polysaccharide chain ordering that effects charge exposure is of high importance for rational design of novel materials. Probing the conformational state of such flexible matter as polysaccharides is a challenging task to which computer methods contribute significantly revealing the statistically relevant cases of both individual molecule conformation in solution and their assembly [60]. Among promising experimental methods to probe the polysaccharide state with high resolution, along with AFM microscopy, a fascinating tool should be mentioned which studies the conformational landscape of an oligosaccharide molecule, sampled by exciting a gas-phase molecular conformer to an ensemble using soft molecule–surface collision [61]. Despite the atomistic depicture of the polymer structure through these methods, the detection of the structure in molecular complexes in soft matter remains challenging. FTIR-spectroscopy is one of the non-destructive methods sensitive to the conformational state of both polysaccharides and proteins, which is able to observe the conformations of both species in gels in situ. It is shown in the next section that the method is highly informative for protein–carrageenan gels where both components have remarkable conformational changes.

## 3. Protein–Polysaccharide Gels

In recent decades, protein–polysaccharide gels have attracted attention due to the broad variability of the tuning gel properties [62]. Other strategies to tune the carrageenan gel structure and properties include the use of sugar and polyols as cosolvents [63] or the admixture of other polysaccharides [64].

Protein–polysaccharide gels can be formed in mixtures of gelling polysaccharides and non-gelling proteins [65], gelling proteins and non-gelling polysaccharides [66], or when both agents gel [67]. Depending on the chemical structure of the biopolymers and the environmental conditions, the gel network is formed due to interpenetration, phase-separation, or coupling of components [68,69]. In the interpenetrating network, two independent gel networks are formed where each network is made by the individual biopolymer [70,71]. The phase-separated network is formed in segregative conditions upon demixing of protein and polysaccharide, the resultant structure of such gels is determined by the rate of coarsening and crosslinking of the gelling components. Since segregation is stopped by gelling, the final morphology depends on the relative rates of these processes [72,73]. The coupled gel network forms in associative conditions when two biopolymers interact with formation of modified junction zones. Often both mixing and demixing behavior is electrostatically driven [74,75]. Below we provide a non-exhaustive list of case studies of protein–carrageenan gels formed upon segregative and associative processes.

The ability of proteins to form binary gels with polysaccharides depends on pH, ionic strength, and type of salt, that determine the balance between attractive and repulsive forces. Eleya and Turgeon [76] reported that β-lactoglobulin, one of the whey proteins, being co-gelled with κ-carrageenan in the course of heating, demonstrates the pH-depending behavior. Above the protein pI, in the region of pH 5–7, the rheological behavior of binary gels upon their formation and melting was attributed to the presence of a phase-separated type of network. While at pH 4.0, below the protein pI, their behavior implies the association of biopolymers with the formation of one continuous network, which significantly enhances the protein gel strength. Croguennoc et al. [77] concluded that at pH 7 and 0.1 M NaCl modest concentrations (up to 1 g/L) of κ-carrageenan accelerate the growth of protein aggregates and gel formation without changing the structure of these aggregates. Further, Ako and co-authors [78] studied the influence of polysaccharides on the β-lactoglobulin aggregation morphology upon phase separation. Using confocal laser scanning microscopy, the authors revealed that the cooling down of the system before the β-lactoglobulin gelling leads to a transient micro-phase separation and subsequent aggregation of κ-carrageenan. However, when the κ-carrageenan network formation is inhibited by NaI, a stable micro-phase separation was observed [78]. In addition, Nguyen et al. [79] showed that upon phase separation in the discussed mixtures the polysaccharide competes with protein for cation binding. The addition of a small amount of CaCl_2_ changed the morphology of β-lactoglobulin aggregates from small strands to larger spherical aggregates, whilst the presence of κ-carrageenan strongly reduced these morphological alterations of protein gel aggregation [79].

The formation of a phase-separated gel network was also reported for the mixtures of κ-carrageenan with protein isolates, including whey protein isolate [80] and its hydrolysate [81], pea protein [82,83], soy protein isolate [84], oat protein [85], proteins of milk [86], soymilk [29], salt-soluble meat protein [23,87], etc. In such gels the rheological properties may be intensified in the presence of κ-carrageenan, as it was shown for whey protein-rich systems [80]. Cakir and co-authors [73] studied the rheology and microstructure of whey protein isolate/κ-carrageenan mixed gels. The rheology of mixed gels shifted to continuous phase from protein to κ-carrageenan. Depending on the polysaccharide concentration the microstructure of gels was characterized as protein continuous, bicontinuous, coarse stranded, and κ-carrageenan continuous. The enhancement of strength and firmness of gels at small concentrations of κ-carrageenan was explained by the increased local concentration of proteins due to phase separation, since the microstructure of such gels was characterized by the presence of carrageenan-rich droplets dispersed in a continuous protein-rich matrix. At higher κ-carrageenan concentrations, gels were weaker and less deformable, whilst the microstructure of gels was shifted from the protein continuous phase to the carrageenan continuous phase. It should be mentioned that there exists a significant influence of NaCl concentration on gel microstructure. The presence of ions changes the gel microstructure from stranded to particulate (micro-phase separated) in a concentration dependent fashion [73]. Babaei [88] reported that the enrichment of the binary whey protein isolate–κ-carrageenan gels with CaCl_2_ leads to additional, approximately sixfold, enhancing of gel hardness. However, from the structural point of view, the intermolecular interactions of these three components were detected in the amorphous phase of the gel [88].

Thus, gel formation driven by repulsive forces and phase separation is associated with changes of microstructure to which the rates of demixture and gelling contribute. The microstructure of such gels is rather characterized by spatially separated aggregates of individual components. The apparent changes of gel rheology may be explained by the level of the individual components network intactness and by the increase of the local concentration of components.

When the gel formation is driven by electrostatic attraction the complexation between protein and polysaccharide may lead to a significant alteration of the structure of junction zones. For example, Duran with co-authors [89,90] showed that in quinoa protein–ι-carrageenan gels both biopolymers are synergically responsible for the formation of the gel matrix. The mixtures between the male gonad hydrolysates of scallop *Patinopecten yessoensis* and κ-carrageenan [91] suggest electrostatic interactions involved in the maintenance of the gel network. In their turn, the protein–polysaccharide complexation via electrostatic interactions, hydrogen bonds, and hydrophobic interactions leads to a well-distributed network structure supporting the stronger gel rigidity [91]. The increasing level of ι-carrageenan results in a more compact network of soya meat analogues [92]. Synergistic interactions of κ-carrageenan with milk proteins promote the development of a cross-linked network in whipped cream [27]. The addition of ι-carrageenan to scallop *Patinopecten yessoensis* male gonad hydrolysates can result in a 116-fold increase of storage modulus compared to that of protein alone [93]. The emulsion-based gels of whey protein isolate with κ- and ι-carrageenans increase the strength and elasticity together with increase of carrageenan content; the network coupled with ι-carrageenan was stronger than that with κ-carrageenan [94]. In turn, the influence of λ-carrageenan on the gel strength was higher than that of ι-carrageenan [95]. Furthermore, the gels formed by κ-carrageenan with hagfish slime show synergistic effects by exhibiting high values of elasticity and viscosity [96]. κ-Carrageenan enhances yolk properties via electrostatic interactions with proteins [97].

Molecular interactions between proteins and polysaccharides occur upon coacervate formation in solution. FTIR- [98] and NMR-spectra [99] can detect the complex formation between charged biopolymers. Le and Turgeon [100] proposed the following mechanism of associative gelation in the β-lactoglobulin and xanthan gum mixtures [62]. The gelation starts by establishing interactions between negatively charged groups of polysaccharide and positively charged moieties of protein with the formation of soluble complexes. Then, along with the change of surface charge of these soluble complexes, interpolymer association occurs where one protein molecule may interact with several polysaccharide chains forming junction zones. This cross-linking process continues further until the completion of the sol-gel transition [100].

Protein–polysaccharide complexation in the cross-linked gel network as well as in coacervates may result in conformational changes in both the protein and polysaccharide, which, in turn, can be one of the factors for the modified gel rheology.

However, despite the fact that the analysis of gel microstructure has accompanied most research dealing with the properties of binary gels, the information about their nano-scale structure and conformational adjustment of counter-parts in complexes is rather scarce.

To have a vision of protein conformation alteration upon binary protein–polysaccharide coacervate formation, some spectroscopic techniques are informative. Several studies report on significant changes in protein secondary structure content. Siddiqui and co-authors [101] reported that purified almond cystatin mixing with λ-carrageenan at 25 °C showed a significant shift in the FTIR-peak intensity, which depicts the helix-to-β-sheet change in the secondary structure of cystatin. The CD spectra confirmed the helix to β-sheet transition. Tang and co-authors [102] observed a decrease of the relative content of α-helixes and β-turns and an increase of β-sheets and random coils in salted duck egg white with the addition of κ-carrageenan. According to the Raman spectroscopy data, a small content of κ-carrageenan promoted the conformational transition of surimi protein from α-helix to β-sheet [103]. In contrast, Zheng and co-authors [104] observed none-to-minimal shift of the β-sheet and the α-helical protein structure in the gels of κ-carrageenan with krill protein isolate. A tendency of globular protein structure to shift from order to disorder was concluded on the basis of low-temperature DSC-curve shift and CD-spectra of lysozyme–κ-carrageenan gels [65].

The conformation of both protein and polysaccharide may be influenced by its counter-part. For example, the unwinding of the ι- and κ-carrageenans double helix was reported to be induced by β-casein [105]. This unwinding is due to preferable electrostatic interaction with the coil polysaccharide chain since the conformational change is fully reversible in conditions of sufficiently high ionic strength [105].

Apparently, in a gel with coupled network the conformational adjustment of polymers, together with the concentration of polymers, pH, and ionic strength, becomes a factor of tunable gel rheology. Then the rational design of the binary composition requires a deep fundamental understanding of structural adjustment upon complex formation. The lack of information about the mutual conformational influence of protein and polysaccharide determines, in particular, the lack of appropriate methodology. CD-spectroscopy, widely useful for protein and polysaccharide conformation probing in solution [106], is not appropriate for gels. Suffering from light scattering, it results in distorted spectra of colloid particles that can mislead the analysis. FTIR-spectroscopy may work with the samples of any phase states, if the characteristic bands of protein and polysaccharides do not overlap (not critical if any) and is suited perfectly to probe simultaneously the conformation of protein and polysaccharide in binary gels in situ. Below, some examples of the application of FTIR-spectroscopy in probing the protein–polysaccharide mutual conformational adjustment in protein–κ-carrageenan gels are given.

### 3.1. κ-Carrageenan–Gelatin Gels

Gelatin is derived from a fibrillar protein collagen and widely used as a gelling agent in food and many technological applications. It forms a thermoreversible gel at ambient temperature due to the capability of its chains to intertwine in a form of triple helix.

The addition of a proper amount of κ-carrageenan to gelatin led to composite gel formation with better strength, toughness, and chewiness [107]. The admixture of κ-carrageenan to gelatin gel is accompanied by non-linear rheology changes as a function of the polysaccharide concentration. At low κ-carrageenan concentration, below the 0.1 mass ratio carrageenan-to-gelatin (the concentration of polymers was kept as 1 wt%), no changes were observed. However, above this threshold ratio the gel elasticity increases, demonstrating a synergistic effect of the polysaccharide and the protein. The secondary structure content in both protein and polysaccharide revealed the molecular reasons underlying the rheology tuning [108].

The FTIR-spectroscopy in a combination with molecular modeling is a powerful tool for invasively probing the structure of both protein and κ-carrageenan. In the FTIR-spectra, the structural bands of protein and polysaccharide do not overlap, so that the influence of one component of mixture to the other one is seen in the spectrum as the band’s parameter changes (e.g., shift or broadening).

**Figure 3:**
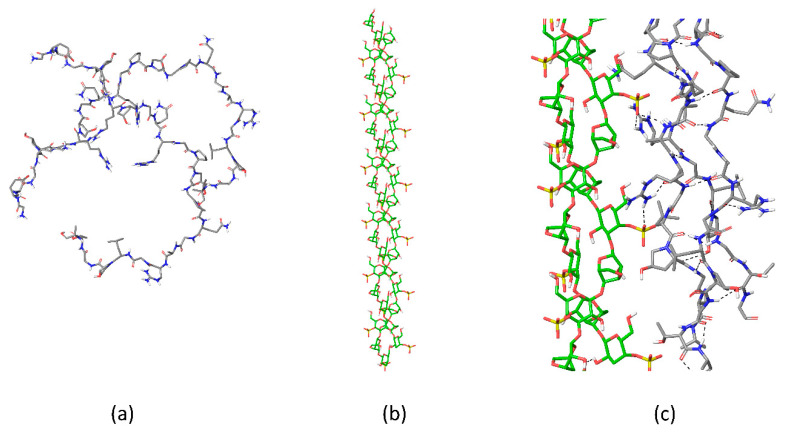
The scheme showing the gelatin chain (**a**) structuring upon interactions with helical κ-carrageenan, (**b**) protein–polysaccharide interactions in the electrostatically coupled complexes, and (**c**) in junction zones of gelatin–κ-carrageenan gels. Color coding: C atoms of protein–grey, C atoms of κ-carrageenan–green, S atoms–yellow, N atoms–blue, O atoms–red, polar H atoms–white, non-polar H atoms not shown for clarity.

In the κ-carrageenan–gelatin mixture below, in the threshold mass ratio, there exists only a slight shift of protein bands together with the polysaccharide ones pointing to chain–chain interactions mediated via electrostatic interactions. Above the threshold value the changes in the thin structure of Amide I indicate the increase in the triple helices content in the protein upon κ-carrageenan admixture. Strikingly, starting with the same mass ratio and above it the content of κ-carrageenan helices increased. Notably, such a polysaccharide concentration in solution without protein was not sufficient for κ-carrageenan gel formation. Such a mutual influence of components in the mixture on the counterpart ordering implies the basis for the formation of junction zones of the novel type, where the helixes of protein and polysaccharide may form ordered bunches (Figure 3).

Comparing the structural results with the rheological data it was concluded [108] that protein–polysaccharide interaction per se did not tune the gel elasticity. The most important factor is the structural changes related to the rise in ordered structures and consequent bunch formation.

The increase of the strength and hardness of the gelatin gels was also reported in the systems of κ-carrageenan and fish gelatin [109]. However, the addition of salts had a negative effect on the rheological behavior of the gel; the low concentrations of NaCl and Na_2_SO_4_ increased the fluorescence intensity by unfolding of the fish gelatin molecules and decreased α-helix and β-sheet contents by destroying the hydrogen bond net between the fish gelatin and κ-carrageenan.

### 3.2. κ-Carrageenan–Lysozyme Gels

Globular proteins also may crosslink carrageenan chains to form gel. Even at low polysaccharide concentrations when polysaccharide itself cannot form a spatial gel network, its interactions with protein may lead to chain crosslinking. In solution, the tendency of polyelectrolytes to saturate the electrostatic interactions results in protein–polysaccharide coacervation with eventual change compensation. There are numerous reports in the literature that show the interaction of globular proteins with sulfated polysaccharides such as heparin [110] and carrageenans [65] results in coacervation. Such a complexation was reported to be accompanied by a rise in the protein’s β-sheet content.

Our results on the interactions of small globular proteins (hen white-egg lysozyme, RNAse from *Bacillus Intermedius*) with κ-carrageenan in gels support these conclusions and highlight the protein–polysaccharide interactions from the point of view of polysaccharide structure importance [111]. Irrespectively of polysaccharide concentration and its initial conformation, its admixture to protein solution results in coagulation and formation of macroscopic clots. However, the conformation of trapped protein depends strongly on polysaccharide conformation and the resultant sulfate groups orientation.

**Figure 4:**
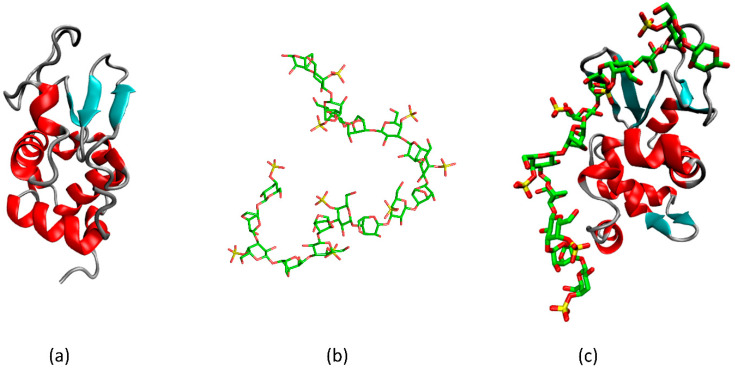
The scheme showing the structural adjustment of hen egg white lysozyme upon interactions with (**a**) κ-carrageenan in protein–polysaccharide gel: native protein structure, (**b**) random coil of κ-carrageenan, (**c**) lysozyme with increased β-sheet content in complexes with κ-carrageenan. Color coding: α-helix–red, β-sheet–cyan, C atoms–green, S atoms–yellow, O atoms–red, H atoms not shown for clarity.

Upon interactions with the flexible κ-carrageenan chain, complex formation with lysozyme occurs at a stoichiometry of 12 disaccharide units per one protein molecule. Interestingly, a similar stoichiometry (mass ratio 1:0.3) was found for lysozyme–λ-carrageenan complexes, which indicates that not all of the sulfate groups in the latter are available for contacts with protein [65,112]. In such complexes (Figure 4), the κ-carrageenan molecules retain their coil conformation. In contrast, a notable structural reorganization in the protein molecules takes place. In a mixed gel, the protein becomes enriched with intramolecular β-sheets. The thin protein structure analyzed by FTIR-spectroscopy revealed that the increase in β-sheets is at the expense of coil content. A similar behavior of oyster protein, the formation of β-sheets, was reported in its gels with κ-carrageenan [113].

**Figure 5:**
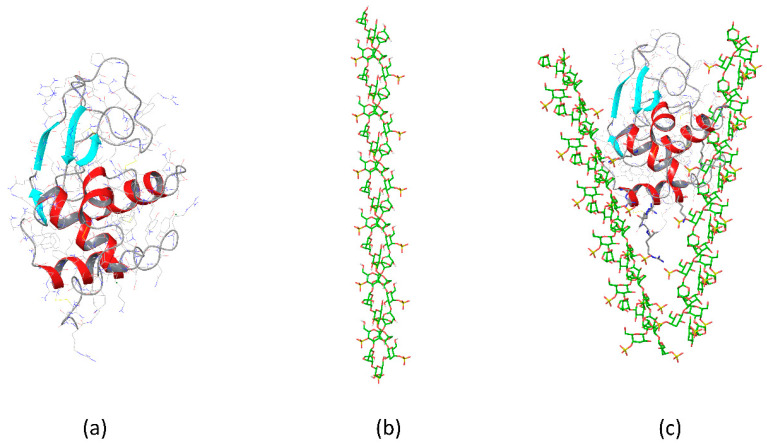
The scheme showing the hen egg-white lysozyme complexes with ordered κ-carrageenan in protein–polysaccharide gel: (**a**) native protein structure, (**b**) double helix of κ-carrageenan, (**c**) lysozyme complexed with two double helices of κ-carrageenan. Color coding: α-helix–red, β-sheet–cyan, C atoms of protein–grey, C atoms of κ-carrageenan–green, S atoms–yellow, N atoms–blue, O atoms–red, H atoms not shown for clarity.

Upon interactions with the stiff κ-carrageenan chains in the form of a double helix, the stoichiometry in the sediment changes for 21 disaccharide units per protein molecule (revealing that in helix less sulfate groups are available to the protein than those compared to the carrageenan coil). The polysaccharide secondary structure preference was regulated by the concentration of κ-carrageenan. In the gel, the polysaccharide retained its predominant helical conformation and the protein remained in its native-like conformation (Figure 5). Interestingly, on mixing the protein and polysaccharide solutions at higher temperatures, when carrageenan is in its coil conformation, the polysaccharide may retain a larger amount of protein. The polysaccharide-to-protein stoichiometric ratio was 14 disaccharide units per protein molecule. Nonetheless, the cooled sediments revealed that κ-carrageenan is helical and the protein remains native-like (according to FTIR-spectra). This reflects the key role of polysaccharide conformation in the protein structure reorganization in binary complexes. The polysaccharide conformation determines the spatial orientation of both sulfate groups and hydrophobic patches of polysaccharide regulating their accessibility to protein. The results of molecular modelling support this conclusion revealing different epitopes on the protein surface binding the κ-carrageenan as a rigid double helix and as a flexible chain. Namely, lysozyme interacts with the double helix of κ-carrageenan via its α-domain and via β-domain upon binding the single carrageenan chain. Interestingly, fragment-based molecular docking together with advanced protein structure sampling using accelerated molecular dynamics showed that binding of flexible κ-carrageenan single chain by β-domain of lysozyme becomes more energetically favorable when the protein is partly desaturated, gaining extra content of β-sheets in the β-domain, which occurs at the expense of the coil [111].

### 3.3. Gels of κ-Carrageenan-Lysozyme Fibrils

Carrageenans are often considered as GAG-mimetics. Being sulfated polysaccharides, similar to carrageenans, glycosaminoglycans have a great number of functions in mammalian organisms. Some of them are in conjunction with long-range interactions with proteins having a potential synergy on the interaction of several chains. However, obtaining structural information about the protein–GAG interaction is challenging, the quantity of spatial structures in PDB was only 105 complexes up to September 2020 [114]. In addition to intercellular signaling mediation, the glycosaminoglycans are involved in the regulation of protein amyloid aggregate formation which causes neurodegenerative disorders such as Alzheimer’s, Parkinson’s, and prion diseases. Such aggregates are made by the stacks of β-reach proteins in which the glycosaminoglycans, i.e., heparan sulfates, are included [115,116,117,118].

Since the drugs and therapy against neurodegenerative diseases are of high demand, fundamental understanding of how polysaccharide influences protein fibrils is needed. Currently, such information is rather contradictory. Presumably, the reason is the use of different proteins in certain studies, the action of a polysaccharide to which, is barely comparable.

We explored the effect of a wide number of polysaccharides on the integrity of fibrils from hen white-egg lysozyme widely used as a model [119]. The tendency was revealed of the linear anionic polysaccharides to induce structural reorganization of β-rich proteins assembled into protofibrils and a possibility of full protein renaturation (unpublished data). In the study we used polysaccharides of full length, the protein molecules, being apparently detached from the protofibril, tended to crosslink polysaccharide chains with eventual gel formation. This implies that short oligosaccharides are promising for the destabilization of amyloid protofibrils as chaperons for further protein renaturation. The broader discussion of a possible role of anionic synthetic polymers capable of recognizing unfolded proteins and to act as chaperons can be found in the following reviews [120,121].

Due to hierarchical spatial organization of amyloid fibrils their assembly may occur in the form of gels [122]. Polysaccharides are used for reinforcement of hydrogels based on fibril structures [123]. Furthermore, both strategies, namely the polysaccharide-induced fibrillation with consequent gel formation and polysaccharide admixture to the gels made of protein fibrils, require basic understanding of interaction-induced conformational transitions in biopolymers, to which FTIR-spectroscopy and molecular modeling may contribute significantly.

## 4. Conclusions

The development of novel materials mimicking biological tissues is of both fundamental and practical interest. Extracellular matrix, mainly consisting of protein and polysaccharide network, retains tissue integrity and takes part in the regulation of cell growth. Therefore, the gels, mimicking these properties in vitro, were proposed as scaffolds for cell growing and to create artificial tissues for regenerative medicine. Gels with tunable mash size are also offered as a material for drug delivery with controlled release. Therefore, molecular instruments for case-dependent tight regulation of crosslinking and rheological properties are needed. The protein–polysaccharide gels show one of the possible ways for tuning the gel microstructure and its firmness. Such gels offer a variety of component compositions and give the temptation even to increase the number of components which is inspired by natural tissues. At the current stage of research, the basic understanding of the structure-property relationship in such gels and the mechanisms, the navigation of this relationship is of high demand for the rational design of material with smart properties. The understanding of the structural states of components in such gels and of the general principles of conformational adjustment upon interactions of components in such complex materials, gives a key for unraveling the structural-property management. The provided examples demonstrate that protein–polysaccharide gels can be based on fibrillar proteins, globular proteins, amyloid fibrils, while the conformational response of these proteins depends on many parameters including polysaccharide conformation. At present the generalization of conclusions is far from being achieved. Nonetheless, the vision of protein–polysaccharide interactions in certain molecular systems gives ideas of how the regulation of external parameters can influence the structure and properties of gels. From the understanding of the rules of the organization of binary protein–polysaccharide systems the complexity may be increased to multicomponent systems and then, in future perspectives, to the realization of molecular interplay in natural tissues.
